# Spin-Coated *vs.* Electrodeposited Mn Oxide Films as Water Oxidation Catalysts

**DOI:** 10.3390/ma9040296

**Published:** 2016-04-19

**Authors:** Simelys Hernández, Carminna Ottone, Sara Varetti, Marco Fontana, Diego Pugliese, Guido Saracco, Barbara Bonelli, Marco Armandi

**Affiliations:** 1 Applied Science and Technology Department, Politecnico di Torino, C.so Duca degli Abruzzi 24, Turin 10129, Italy; carminna.ottone@pucv.cl (C.O.); sara.varetti@polito.it (S.V.); marco.fontana@polito.it (M.F.); diego.pugliese@polito.it (D.P.); guido.saracco@polito.it (G.S.); barbara.bonelli@polito.it (B.B.); marco.armandi@polito.it (M.A.); 2 Center for Space Human Robotics, IIT@POLITO, Istituto Italiano di Tecnologia, C.so Trento 21, Turin 10129, Italy; 3 School of Biochemical Engineering, Pontificia Universidad Católica de Valparaíso, Avenida Brasil 2147, Valparaíso 2362803, Chile

**Keywords:** water oxidation, electrodeposition, manganese oxides films, polyethylene oxide, electrochemical impedance spectroscopy

## Abstract

Manganese oxides (MnO_x_), being active, inexpensive and low-toxicity materials, are considered promising water oxidation catalysts (WOCs). This work reports the preparation and the physico-chemical and electrochemical characterization of spin-coated (SC) films of commercial Mn_2_O_3_, Mn_3_O_4_ and MnO_2_ powders. Spin coating consists of few preparation steps and employs green chemicals (*i.e.*, ethanol, acetic acid, polyethylene oxide and water). To the best of our knowledge, this is the first time SC has been used for the preparation of stable powder-based WOCs electrodes. For comparison, MnO_x_ films were also prepared by means of electrodeposition (ED) and tested under the same conditions, at neutral pH. Particular interest was given to α-Mn_2_O_3_-based films, since Mn (III) species play a crucial role in the electrocatalytic oxidation of water. To this end, MnO_2_-based SC and ED films were calcined at 500 °C, in order to obtain the desired α-Mn_2_O_3_ crystalline phase. Electrochemical impedance spectroscopy (EIS) measurements were performed to study both electrode charge transport properties and electrode–electrolyte charge transfer kinetics. Long-term stability tests and oxygen/hydrogen evolution measurements were also made on the highest-performing samples and their faradaic efficiencies were quantified, with results higher than 95% for the Mn_2_O_3_ SC film, finally showing that the SC technique proposed here is a simple and reliable method to study the electrocatalytic behavior of pre-synthesized WOCs powders.

## 1. Introduction

One of the most promising approaches for the exploitation of solar energy is the production of fuels by means of photocatalytic processes. Water splitting (WS) is an interesting technology to transform solar energy and to store it in the form of green chemicals or fuels, such as oxygen and hydrogen. The overall reaction (1) is the sum of the two half-reactions of oxidation (2) and reduction (3):

2 H_2_O → 2 H_2_ + O_2_ (1)

2 H_2_O → O_2_ + 4 H^+^ + 4 e^−^ (2)

4 H^+^ + 4 e^−^ → 2 H_2_ (3)

The half-reaction of water oxidation (WO) is the bottleneck step, not only for H_2_ production but also in the systems involving the reduction of CO_2_ for the production of other fuels or chemicals [1]. The limitation of such uphill reaction is due to the formation of a double O-O bond and the release of four protons and four electrons, which require high overpotentials. According to the literature, the best water oxidation catalysts (WOCs) are RuO_2_ and IrO_2_ [2], endowed with high catalytic activity, good stability and conductivity. However, their use is limited by their high cost, since they are based on precious and rare metals. Therefore, the development of WOCs based on non-toxic and inexpensive materials, present in abundance on Earth and working under accessible reaction conditions, is an up-to-date research topic. Recently, Mn-based catalysts have attracted much interest as WOCs [3]. Many literature works proposed different Mn-based species, starting from the wide variety of manganese oxides (MnO_x_) polymorphs [4,5,6,7] to the synthesis of complex molecules [8]. In some cases, the incorporation of Ca into the structure has the aim of mimicking the metal core (*i.e.*, Mn_4_CaO_5_) that catalyzes WO reaction in photosynthetic systems [7,9,10].

The practical application of a WOC in a water splitting (WS) device involves, in most cases, the use of a photoelectrochemical cell (PEC). To this end, Swierk and Mallouk proposed a dye-sensitized PEC [11], in which the anode is a semiconductor (*i.e.*, TiO_2_ nanoparticles) coupled to a dye and a WOC anchored to the dye-sensitized TiO_2_ surface. In such a system, the three different species are able to electronically interact amongst themselves, and guarantee a proper charge transport due to the smart molecular engineering of the electrode. Regarding the semiconductors used in photoanodes, many pure and modified photocatalysts (*i.e.*, TiO_2_ [12], ZnO [13], Fe_2_O_3_ [14], BiVO_4_ [15]) have been deeply studied in photoelectrochemical systems, with the goal of optimizing their performances in WS. However, the electrochemical (EC) study of single WOCs is limited in the literature. WOCs are usually synthesized in powder form, and are studied in heterogeneous reactors in the presence of sacrificial oxidants [4,16,17,18,19], because this is a simple and acknowledged method to identify their catalytic properties and potentialities. However, the operational conditions (*i.e.*, fluid dynamics, electronic transport, incidence of illumination) of such systems are completely different with respect to the electrochemical ones, and therefore are not useful for an applicative extrapolation to PEC devices.

The deposition of a WOC on a conductive electrode allows determining important properties and establishing the performance of the material in conditions close to an actual WS device. As far as the preparation of MnO_x_-based electrodes is concerned, the most common method is the electrodeposition (ED) of thin films [20]. Such approach allows a good control of the quantification of the deposited MnO_x_, which is directly related to both deposition time and applied current. However, in the absence of post-deposition annealing treatments, ED generally leads to an amorphous material rather than a crystalline film [21,22,23].

On the other hand, the production of stable films from powder materials remains an issue. Recently, the screen-printing method has been used to prepare micrometer-thick layers of pre-synthesized calcium manganese oxide [24] and β-MnO_2_ [25] particles into a transparent conductive oxide (*i.e.*, fluorine-doped tin oxide, FTO).

In this work, we propose the fabrication of films from WOC powders by a simple spin-coating (SC) approach, which involves few preparation steps and green chemicals (*i.e.*, ethanol, acetic acid, polyethylene oxide or PEO and water). SC is widely used at both lab and industrial scale for the manufacturing of semiconductor films [26,27]. It has the advantage of producing homogeneous and reproducible films when combined with sol-gel processes [28], however it has not been used, so far, to prepare films of pre-synthesized WOC particles, most probably because it requires some modifications in order to be reproducible. Moreover, SC has the advantage, with respect to screen-printing, of requiring the use of a small amount of material for the fabrication of the WOCs films, therefore reducing fabrication costs and allowing the screening of lab-scale prepared materials. The use of Nafion (which is commonly employed as a binder in electrode preparation) was discarded, in order to elude problems related to its low conductivity (that could hinder electro-activity measurements), its swelling in aqueous media and the absence of porosity (that in most of the cases causes the film detachment after long-time operation). As an alternative, the use of polyethylene oxide (PEO) as an additive has the function of increasing the viscosity of the MnO_x_-containing suspension, also acting as a binder and allowing particles to distribute evenly and increase their adhesion to the substrate, yielding to a homogeneous film. Other advantages of PEO are the non-toxicity, the low decomposition temperature (~200 °C for PEO with molecular weight (M_w_) of *ca.* 10^6^ g·mol^−1^ [29]) and the high water solubility, which render the process greener because it avoids the use of high quantities of flammable solvents. Besides, the broad variety of available M_w_ of PEO (from 300 to 10^7^ g·mol^−1^) could allow a proper tuning of the suspension viscosity, and thus of the final film thickness, rendering the process a very versatile one. The SC method is moreover mild enough to retain the chemical characteristics of the applied MnO_x_ phases and, hence, it could be used to produce films starting also from other WOC powders, in order to study their electrocatalytic behavior.

Three commercially available MnO_x_ powders (*i.e.*, MnO_2_, Mn_2_O_3_, Mn_3_O_4_) were employed as model materials of pre-synthesized particles, in order to evaluate their electrochemical behavior towards WO reaction and prove the applicability of the new deposition method here presented. In addition, the phase transition of the commercial MnO_2_ powder to α-Mn_2_O_3_ [30] was promoted at 500 °C, in order to compare the performance of films having the same crystalline phase (*i.e.*, α-Mn_2_O_3_), but different crystal sizes and morphologies. Both electrocatalytic and electronic properties of SC films were compared to those of ED films. The so obtained MnO_x_ electrodes were tested by cyclic-voltammetry in dark conditions, Tafel analysis and chrono-amperometry with simultaneous O_2_ and H_2_ evolution measurement, with the aim of critically analyzing their electrochemical performance. Besides, on the most performing films electrochemical impedance spectroscopy (EIS) measurements were used to quantify both electrodes charge transport properties and electrode-electrolyte charge transfer kinetics, and to discuss their influence on the final electro-activity of the investigated WOCs.

## 2. Materials and Methods

### 2.1. Materials

All chemicals (H_2_SO_4_, H_2_O_2_, Mn(CH_3_COO)_2_·4H_2_O, polyethylene oxide (PEO, M_W_ = 10^6^), Na_2_SO_4_, NaH_2_PO_4_·2H_2_O and Na_2_HPO_4_·H_2_O) and commercial manganese oxides (Mn_2_O_3_, Mn_3_O_4_, and activated porous MnO_2_) were purchased from Sigma-Aldrich (Munich, Germany).

### 2.2. Films Prepared by Spin-Coating

MnO_2_ powder (with specific surface area, SSA, of 98 m^2^·g^−1^) was used without any pre-treatment. Instead, both Mn_2_O_3_ (SSA = 2 m^2^·g^−1^) and Mn_3_O_4_ (SSA = 0.6 m^2^·g^−1^) were mechanically ground for 20 h at 800 rpm before use, by means of a planetary micro mill (Pulverisette 7, Fritsch, Idar-Oberstein, Germany) working with 10 stainless steel balls (10 mm in diameter). After grinding, the particle diameter was reduced (Section 3.1) and the SSA increased up to 14 m^2^·g^−1^ and 10 m^2^·g^−1^ for Mn_2_O_3_ and Mn_3_O_4_ powders, respectively [30]. Films of such MnO_x_ powders were made by spin-coating a suspension prepared by modifying a procedure that was previously employed for the fabrication of efficient ZnO-based photoelectrodes [31]. In a typical preparation, 0.2 g powder were added to a solution of ethanol and acetic acid (20:1, *v*/*v*) and then mixed with 313 mg of an aqueous solution of PEO (3 wt%) in an ultrasound bath for 15 min. 150 μL of the suspension were then poured on a FTO-coated glass substrate (2 × 2 cm^2^), previously cleaned with piranha solution (H_2_SO_4_:H_2_O_2_ = 3:1, *v*/*v*). The coating was performed with a 2 steps spinning program, first at 500 rpm and then at 2000 rpm, with a duration of 10 s for each step and an acceleration of 100 rpm·s^−2^. The films were subsequently dried at 90 °C for 30 min in order to remove both ethanol and acetic acid. Finally, the removal of PEO (an insulator) at a temperature higher than 200 °C is required in order to appropriately evaluate the electrochemical behavior of the deposited MnO_x_ material. Thus, selected samples were calcined at 400 °C for 2 h in static air at a heating rate of 5 °C·min^−1^, also with the aim of increasing the adhesion between the film and the substrate. Only the MnO_2_ sample (amorphous) was calcined at higher temperature (*i.e.*, 500 °C), in order to induce changes in the crystalline phase to α-Mn_2_O_3_ [30]. The samples are named considering the method used to achieve the film and the crystal phase. The MnO_2_ sample will therefore be referred to as Mn_2_O_3_-SC-TT.

### 2.3. Films Prepared by Electrodeposition

The preparation of the electrodeposited films was adapted from the literature [22]. A typical three-electrodes cell, with a Pt wire as counter electrode, Ag/AgCl as a reference electrode and a FTO-coated glass substrate as the working electrode, was used for the electrodeposition of amorphous MnO_x_ films. The FTO substrates were previously cleaned with piranha solution (H_2_SO_4_:H_2_O_2_ = 3:1, *v*/*v*) and masked to leave an active surface of 2 × 2 cm^2^. The electrodeposition was performed at constant current of 1 mA for 3 different deposition times *t* (ED*1*, ED*5* and ED*10* correspond to *t* = 1 min, 5 min and 10 min, respectively), in a solution containing 0.1 M Na_2_SO_4_ as supporting electrolyte and 0.1 M Mn(CH_3_COO)_2_·4H_2_O as Mn precursor. The pH was adjusted to 5.7 with acetic acid. The films were tested both as-synthesized and after calcination at 500 °C for 2 h (with a heating rate of 5 °C·min^−1^) in calm air, with the aim of forming the crystalline phase α-Mn_2_O_3_ (samples referred as ED*tc*, where *t* stays for the deposition time and *c* for the calcination step).

### 2.4. Characterization Techniques

X-ray diffraction (XRD) patterns of the films were collected on an X′Pert Phillips diffractometer using Cu Kα radiation = 1.541874 Å (20–60 2θ range; step width = 0.02 degree; time per step = 2 s) and indexed according to the Powder Data File database (PDF 2000, International Centre of Diffraction Data, Philadelphia, PA, USA). The morphology of the samples was investigated by Field Emission Scanning Electron Microscopy (FE-SEM) (ZEISS, Oberkochen, Germany) on a Zeiss Supra 40 instrument. Cross-section images were used to determine the average thickness of the films. UV-Visible transmittance spectra were recorded by using a UV-Vis Varian Cary 5000 spectrophotometer (Agilent Technologies, Inc., Santa Clara, CA, USA).

### 2.5. Electrochemical Measurements

Electrochemical measurements were run with a standard three-electrodes setup in a lab-made glass cell with an Ag/AgCl (3 M KCl) electrode as reference electrode and a Pt wire as counter electrode. The measurements were recorded by using a multichannel VSP potentiostat/galvanostat (BioLogic), equipped with EC-Lab v. 10.1x software for data acquisition. The reported potentials are referred to the reversible hydrogen electrode (RHE). The measured potentials *vs.* the Ag/AgCl reference electrode were converted to the RHE scale by using the Nernst Equation: $E_{RHE}=E_{Ag/AgCl}+E_{Ag/AgCl}^{0}+0.059\;pH$. *E_RHE_* is the converted potential *vs.* RHE, *E_Ag/AgCl_* is the experimental potential measured against the Ag/AgCl reference electrode, and $E_{Ag/AgCl}^{0}$ is the standard potential of Ag/AgCl (3 M KCl) at 25 °C (*i.e.*, 0.210 V). The catalytic activity of MnO_x_ films was tested at pH = 7.0 in 0.1 M sodium phosphate buffer. Cyclic voltammetries (CVs) were performed in the range between 0.6 and 2.0 V *vs.* RHE with a sweep rate of 20 mV·s^−1^. Current density was calculated by considering the geometrical area of the FTO substrate covered by the MnO_x_ film (~4 cm^2^).

For Tafel plot analysis, current density was recorded stepwise between 1.4 V and 2.0 V *vs.* RHE until reaching quasi-stationary currents. Therefore, the electrode potential was increased by 40 mV steps and held for 5 min. The potential was corrected for the ohmic drop, hence, impedance spectra were recorded at every potential/current step at 10 kHz with a modulation amplitude of 20 mV in order to determine the ohmic resistance of the solution (*R_s_* ~ 40 Ω). The overpotential (η) values reported in both Tafel plot and Table 1 were calculated as follows: $\eta =E_{RHE}-E_{RHE}^{0}-i\;R$, where $E_{RHE}^{0}$ is the standard potential of the water splitting reaction (~1.23 V) and *i* is the current. EIS curves were recorded using the same potentiostat described above from 0.1 Hz to 0.5 MHz, with an alternating current (AC) amplitude of 20 mV at different applied potentials (*i.e.*, 1.0 V, 1.2 V and 1.4 V *vs.* Ag/AgCl).

Oxygen and hydrogen evolutions were measured by a Varian 490 micro-GC equipped with a Molsieve column during chrono-amperometry test at a fixed potential of 2.0 V *vs.* RHE, after purging of the cell with a continuous Ar flow for 1 h. An Argon flow rate of 25 Nml·min^−1^ was used in order to carry the evolved O_2_ and H_2_ gases from the cell to the micro-GC, while the solution was maintained under continuous stirring to increase the mass transfer of the dissolved gases to the gaseous media.

## 3. Results and Discussion

### 3.1. Spin-Coated Samples

The SC method was initially tested with as-purchased micrometric Mn_2_O_3_ and Mn_3_O_4_ powders (average particle size ~2 to 5 μm). Nevertheless, the large particle size (Figure S1 in the Supplementary Materials) led to a poor adhesion of the catalyst to the substrate, causing some material detachment during electrochemical tests, with a consequent drastic decrease in catalytic activity after few voltammetry cycles. Therefore, both Mn_2_O_3_ and Mn_3_O_4_ powders were ball-milled in order to reduce their particle size [30]. SC of ball-milled samples led to a more effective adhesion of the film on the FTO substrate, and allowed for better particle interconnection. In contrast, the commercial MnO_2_ powder was characterized by a heterogeneous particle size distribution, and it was used without any modification. Indeed, during the SC procedure only the smaller particles adhered to FTO (the larger ones being removed by centrifugal force), yielding to stable films.

**Figure 1:**
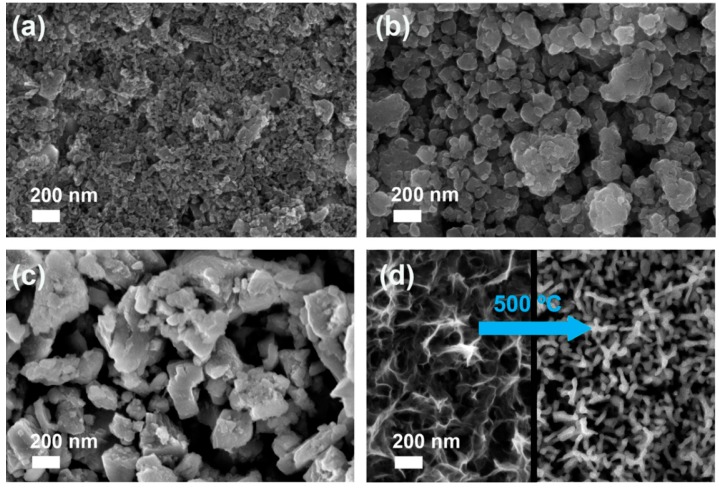
FE-SEM images of a top view of MnO_2_ (**a**); Mn_2_O_3_ (**b**) and Mn_3_O_4_ (**c**) SC films after annealing at either 500 °C or 400 °C; (**d**) FE-SEM images of a top view of the ED film: as-prepared sample (left image) and annealed at 500 °C (right image).

The morphology of SC films of the commercial MnO_x_ powders is shown in the FE-SEM images in Figure 1. The MnO_2_ film is constituted of nearly spherical nanoparticles with average diameter of 20 nm. Concerning Mn_2_O_3_ and Mn_3_O_4_ films, the particles show extremely variable morphology and size; however, it must be stressed that ball-milling was effective in decreasing their size from the original micrometric size (2 to 5 μm) to 30 ÷ 170 nm (Mn_2_O_3_) and 70 ÷ 400 nm (Mn_3_O_4_). With all the considered samples, particles are well-distributed on FTO surface by forming a continuous coverage of the substrate. The film thickness, as measured by cross-section FE-SEM, lies in the 500 ÷ 1500 nm range for all the SC samples (Figure S2 in Supplementary Materials).

**Figure 2:**
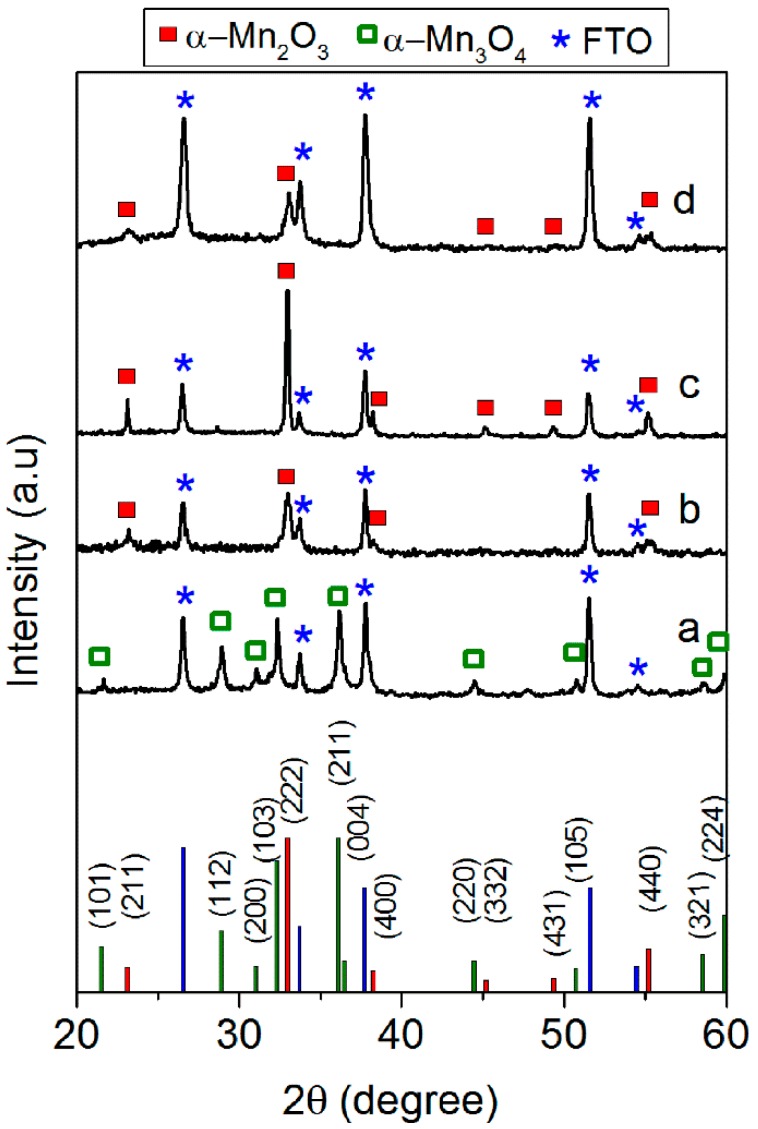
XRD patterns of Mn_3_O_4_-SC (**a**); Mn_2_O_3_-SC (**b**); Mn_2_O_3_-SC-TT (**c**) and ED*5c* (**d**) films. Indices of each crystalline phase found in the XRD patterns are reported in the bottom section of the figure.

According to our previous study [30], the calcination of the MnO_2_ SC film at 500 °C (Mn_2_O_3_-SC-TT) resulted in the formation of an α-Mn_2_O_3_ crystalline phase, as confirmed by the XRD pattern (curve c, Figure 2). The structural reorganization apparently did not affect particle morphology, but led to a decrease of SSA (from 98 to 20 m^2^·g^−1^) [30]. In contrast, the crystalline phases of both Mn_2_O_3_ and Mn_3_O_4_ powders were not affected by the thermal treatment at 400 °C performed on their respective SC films (*i.e.*, Mn_2_O_3_-SC and Mn_3_O_4_-SC). Indeed, the XRD pattern of Mn_2_O_3_-SC film (curve b in Figure 2) evidenced the main peaks (at 2θ of about 23.2° and 32.9°) corresponding to the (211) and (222) crystalline planes of bixbyite. Similarly, the XRD pattern of Mn_3_O_4_-SC film (curve a in Figure 2) showed the typical diffraction peaks of hausmannite.

**Figure 3:**
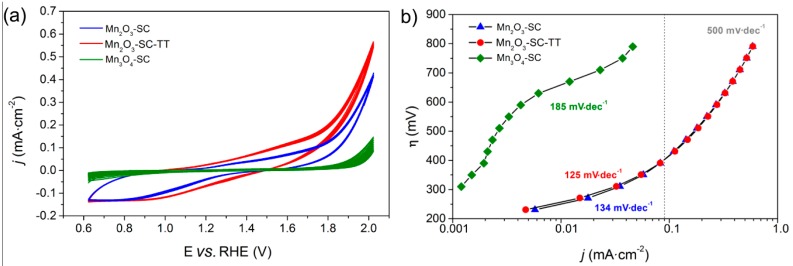
Electrochemical characterization of the SC-films: cyclic voltammograms (**a**) and Tafel plots (**b**) in 0.1 M Na-phosphate buffer (pH = 7.0). Potential (E) is referred to the RHE (see Section 2.5).

The catalytic activity of the MnO_x_ films was evaluated by using them as anodes in the electrochemical WS reaction, with Na-Phosphate buffer as electrolyte (pH = 7.0). The multiple cyclic-voltammetry (CV) curves in Figure 3a evidence the current density behavior of the different materials as a function of the applied potential: the adopted SC procedure yielded to films with good stability, since the 10 CV cycles were highly reproducible for all the samples studied.

As expected, the Mn_3_O_4_-based electrode showed lower current densities and a higher on-set potential (Table 1) for WO than Mn_2_O_3_ based ones, in agreement with the higher WO activity of α-Mn_2_O_3_ [4]. Accordingly, the Tafel plot (Figure 3b) of Mn_3_O_4_-SC film showed a linear η *vs.* log(*j*) dependency only in the high overpotential range (*i.e.*, η = 600 ÷ 750 mV), with a Tafel slope of 185 mV·dec^−1^. On the other hand, the complete transformation of MnO_2_ into α-Mn_2_O_3_ after calcination at 500 °C is confirmed by the similar electrochemical performances in both CV curves and Tafel plots of Mn_2_O_3_-SC-TT and Mn_2_O_3_-SC films. The slightly higher current densities obtained with Mn_2_O_3_-SC-TT in CV curves could be due to its marginally higher surface area and/or to differences in the density and thickness of the film. Notably, our previous work concerning the WO activity of such MnO_x_ powders, as measured by using a sacrificial oxidant (*i.e.*, the Ru(bpy)_3_^2+^/S_2_O_8_^2−^ photosystem) [30], reported a comparable activity (as expressed per unit mass of catalyst) for both Mn_2_O_3_-SC-TT and ball-milled Mn_2_O_3_, definitely higher than those obtained with ball-milled Mn_3_O_4_ powder [30]. It is generally acknowledged that a thorough comparison between photochemical and electrochemical data is rather difficult. Indeed, the former only provide information on the intrinsic activity of the WOC catalytic sites, whereas the latter are affected by different factors such as resistance to charge transport, particle interconnection and their contact with FTO. However, such results are in agreement with the recent work performed by Smith *et al.* [32], where the WO catalytic performances of different MnO_x_ as measured by photochemical and electrochemical assays are compared, confirming as a general trend the superior activity of Mn^3+^-containing catalysts.

The Tafel plots for Mn_2_O_3_-SC-TT and Mn_2_O_3_-SC films (which practically overlap) show two distinct regions, separated by an inflection at *ca.* 400 mV of overpotential. At low overpotential, Tafel slopes of 125 and 134 mV·dec^−1^ were obtained for the Mn_2_O_3_-SC-TT and Mn_2_O_3_-SC films, respectively, the values being comparable to those reported in the literature for that crystalline phase [33]. However, the WO overpotentials of such commercial Mn_2_O_3_ powder films are higher than those already reported for lab-made MnO_x_ [23,25,33]. Indeed, the two samples reached current densities of 0.1 and 0.6 mA·cm^−2^ at about 420 mV and 800 mV of overpotential, respectively. Notably, a drastic positive deviation from linearity is observed in the Tafel plots at high overpotential (>300 mV). This behavior can be attributed to electron transport limitations in the catalyst films at high applied potentials. In order to deeply investigate such phenomena, EIS analysis was performed at different potential values (Section 3.3).

### 3.2. Electrodeposited Samples

Electrodeposition is an advantageous technique to prepare MnO_x_ films, which allows the tuning of the amount of deposited material by varying the deposition time. The formation of MnO_x_ occurs according to Equation (4), where Mn^2+^ ions in solution react with water at the electrode surface, leading to the formation of amorphous MnO_2_.

(4) $${\text{Mn}}^{2+}+2\;{\text{H}}_{2}O\to {\text{MnO}}_{2}+4\;{\text{H}}^{+}+2\;{\text{e}}^{-}$$

The films prepared by ED evidenced a nanosheets shaped morphology (Figure 1d, left side) and an amorphous structure [23]. Indeed, XRD patterns of the as-prepared ED films (not reported) showed only the diffraction peaks due to FTO, regardless of the deposition time. In order to obtain the α-Mn_2_O_3_ phase, a thermal treatment at 500 °C was therefore performed. A change in film morphology (*i.e.*, from nanosheets to nanorods) was induced by thermal treatment, as shown in the FE-SEM image in Figure 1d, right side. The formation of the expected α-Mn_2_O_3_ crystalline phase was confirmed by XRD only for sample obtained with a deposition time *t* ≥ 5 min, showing the characteristic (211) and (222) diffraction peaks of bixbyite (Figure 2d). The film deposited with *t* = 1 min was likely too thin to give a resolved XRD pattern. Such a hypothesis is supported by UV-visible spectra and digital pictures of the ED films (Figures S3 and S4), showing 80% transmittance in the whole visible region only for the sample deposited for 1 min. By increasing the deposition time, the film became darker and transmittance was markedly reduced.

**Figure 4:**
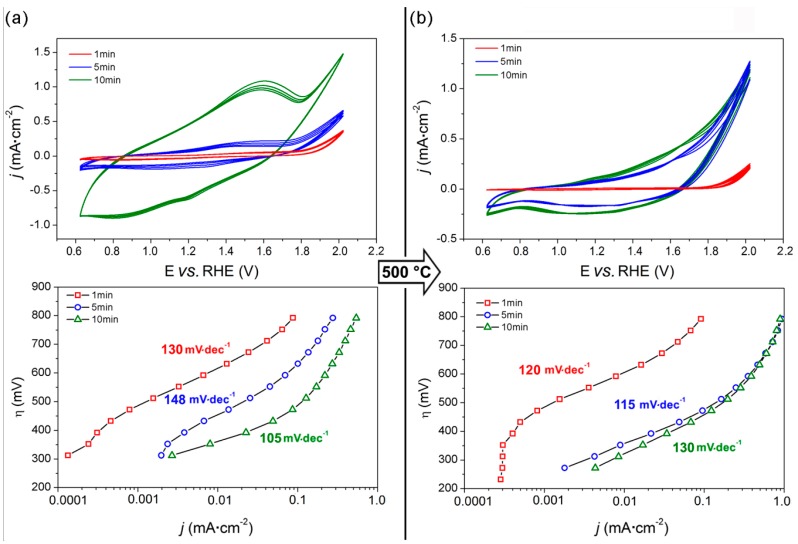
Electrochemical characterization of ED films prepared by electrodeposition at different deposition times: cyclic voltammograms (upper panel) and Tafel plots (lower panel) of the as-deposited (**a**) and calcined (**b**) films in 0.1 M Na-phosphate buffer (pH = 7.0). Potential (E) is referred to the RHE (see Section 2.5).

Figure 4 reports the CV curves and Tafel plots of both as-made and calcined films prepared at different ED times.

The as-made samples (Figure 4a) showed a pseudo-capacitive behavior, typical of MnO_2_ electrodes, particularly pronounced for longer deposition times (*i.e.*, 10 min). Pseudo-capacitance arises from electron transfer at the Mn surface sites, the charge transfer being balanced either by chemisorption/desorption of electrolyte cations or by insertion/deinsertion of protons [34], and thus it is expected to be proportional to the exposed surface area, *i.e.*, to the amount of deposited material. The CVs of the as-made films show also an anodic peak at 1.5 V *vs.* RHE (and two cathodic counterparts at 1.2 and 0.9 V *vs.* RHE), indicating the presence of Mn (II) species which can be oxidized to Mn(III/IV) [33]. In contrast, both the pseudo-capacitive behavior and the redox features characterizing the as-deposited films disappear after calcination at 500 °C, as expected after the formation of the α-Mn_2_O_3_ phase.

The catalytic activity of the as-deposited films increased with the deposition time *t* (ED*1*, ED*5* and ED*10* correspond to *t* = 1 min, 5 min and 10 min, respectively). Indeed, an overpotential of 800, 625 and 500 mV is required to obtain a 0.1 mA·cm^−2^ current density with ED*1*, ED*5* and ED*10*, respectively. In addition, similarly to what is observed for the SC films, the Tafel plots of the ED films show an inflection point, the position of which moves towards lower η at increasing deposition time. A linear η *vs.* log(*j*) dependency in the 470 ÷ 670, 390 ÷ 550 and 315 ÷ 430 mV overpotential range, and the corresponding Tafel slopes of 130, 148 and 105 mV·dec^−1^ are observed with ED*1*, ED*5* and ED*10*, respectively.

After calcination (samples are referred as ED*tc*, where *c* stays for the calcination step, see Figure 4b), the Tafel slopes of both ED*1*c and ED*5c* were lowered to 120 and 115 mV·dec^−1^, respectively, whereas that of ED*10c* increased to 130 mV·dec^−1^. The thermal treatment is particularly beneficial for intermediate deposition time (*i.e.*, for ED*5c*), as shown by the widening towards lower η of the linear portion of the Tafel slope (270 ÷ 500 mV), and by the consequent decrease in η required to obtain a 0.1 mA·cm^−2^ current density *(i.e.*, 470 mV). Interestingly, such an effect is not likewise marked for ED*10c*, suggesting that there is a limit in the α-Mn_2_O_3_ film thickness beyond which the whole exposed surface is not effectively exploited. Curiously, ED*5c* showed a film thickness of the same order of those obtained by SC method (Figure S2).

The Tafel plots of ED*5c* and ED*10c* practically overlap at high potentials, achieving 0.9 mA·cm^−2^ current density at η = 800, *i.e.*, 30% higher than those obtained by the SC films at the same potential. Similarly to what observed for the SC films, the reaction rate with ED films (both as-deposited and calcined) at high overpotential does not increase linearly with the applied potential, probably due to a limitation in the electronic transport within the film.

In conclusion, the optimal compromise between the MnO_x_ deposited amount and the activity in WO reaction was obtained with the sample ED*5c*. For this reason, that electrode was selected for a more detailed study (*i.e.*, EIS and oxygen/hydrogen evolution measurements), together with the SC films having the same α-Mn_2_O_3_ phase and similar thickness.

### 3.3. Charge Transfer and Transport Mechanisms in Mn_2_O_3_-Based Films

**Figure 5:**
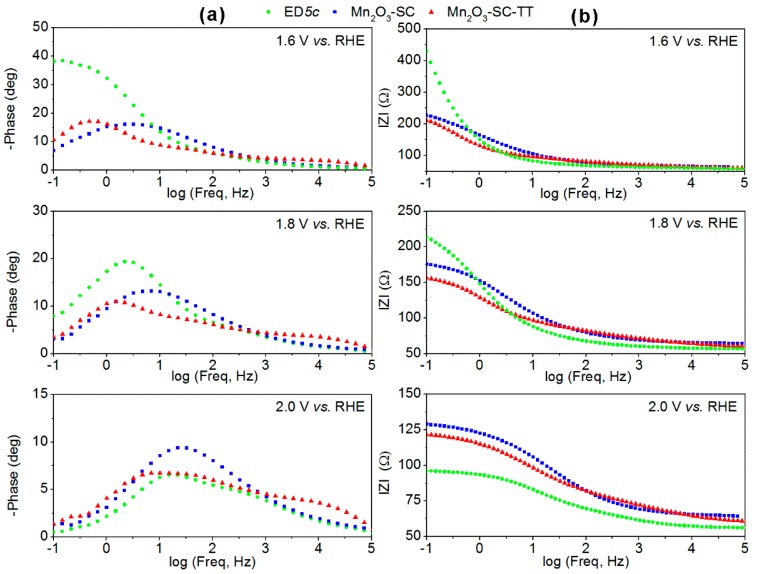
Bode plots representing (**a**) phase and (**b**) module of impedance acquired during EIS measurements by using the α-Mn_2_O_3_-based electrodes at 1.6, 1.8 and 2.0 V *vs.* RHE.

The spin-coated Mn_2_O_3_-SC and Mn_2_O_3_-SC-TT and the electrodeposited ED*5c* films were characterized by EIS at different applied potentials (higher than the on-set potential) at which the electrodes exhibit a current density greater than 0.01 mA·cm^−2^, with the aim of comparing their charge transfer and transport properties. EIS results at 1.6, 1.8 and 2.0 V *vs.* RHE (corresponding to about 380, 530 and 640 mV of overpotential, respectively) are reported in Figure 5 in the form of Bode plots, which show both phase and modulus (|Z|) of impedance *vs.* frequency. Nyquist plots are also reported in Figure S5 of the Supplementary Materials for the sake of completeness.

Concerning the phase spectra in Figure 5a, two features are related to the two different processes occurring in the analyzed electrochemical system: the high frequency peak centered at about 100 Hz, associated with the charge transport properties of the electrode material, and the lower frequency peak, associated with the charge transfer at the electrode/electrolyte interface [28,35]. For all the samples, in the low frequency region (<100 Hz) impedance decreases with the increase of the applied potential, because of an enhancement of the reaction kinetics, which is induced by the electrical forces, but depending on the surface properties of the electrode materials. The same behavior is observed by a reduction of the semicircles diameter at increasing potential values in the Nyquist plots of Figure S5. Indeed, if the |Z| values at 0.1 Hz are considered, the charge transport resistance for ED*5c* decreases of about four times when passing from 1.6 to 2.0 V *vs.* RHE, whereas for the SC films it is halved. Furthermore, it is evident that the ED*5c* film retains better charge transport properties than the SC samples, as lower |Z| values are obtained at *ca.* 100 Hz for all the applied potentials. Such results are in agreement with the CV analysis and Tafel plots, and indicate that both nanostructuration and good adhesion of the ED film (which was directly grown on the FTO-glass substrate) play an important role in the increase of the current density with the applied potential. Furthermore, EIS analysis explains the higher electrocatalytic activity of ED*5c* electrodes at high potential (*i.e.*, 2 V *vs.* RHE).

It is of note that at low overpotential (*i.e.*, 380 mV or 1.6 V *vs.* RHE) the SC films presented faster kinetics at the electrodes-electrolyte interface, showing half of the charge transfer resistance with respect to the ED film. Such result suggests that the SC films retain a more defined crystalline structure and, therefore, have better-defined electrocatalytic properties at low applied potentials than the ED film. On the other hand, the highly heterogeneous adhesion to the substrate and the interparticles contact within SC films have an influence on the maximum current densities that can be achieved.

Finally, the Mn_2_O_3_-SC-TT film apparently evidenced faster kinetics than the Mn_2_O_3_-SC film, as observed from slightly lower |Z| values. Nevertheless, its higher phase values in the high frequency region (~1000 Hz) indicate a pseudo-capacitive effect and suggest that some residual MnO_2_ phase could still be present in this sample.

### 3.4. Oxygen/Hydrogen Evolution Measurements

**Figure 6:**
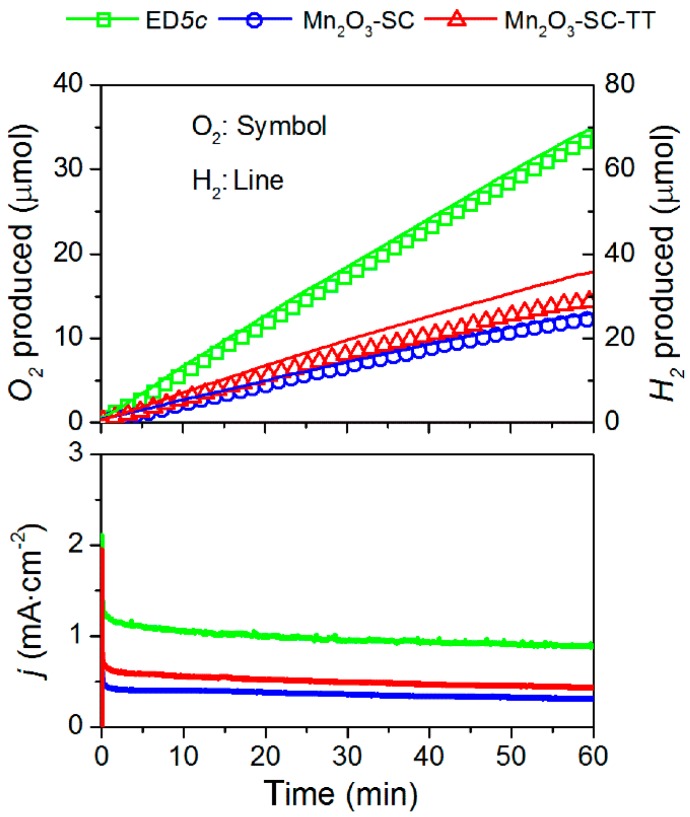
Oxygen and hydrogen evolutions under an applied potential of 2.0 V *vs.* RHE. Time course of the O_2_ and H_2_ production analyzed by a micro-GC (**up**) and of the measured current densities (**bottom**).

The α-Mn_2_O_3_-containing electrodes (Mn_2_O_3_-SC, Mn_2_O_3_-SC-TT and ED*5c*) were selected and tested under the same chrono-amperometric conditions (*i.e.*, 2.0 V *vs.* RHE for 1 h) in order to verify their long-term stability and their faradaic efficiencies in O_2_ and H_2_ evolution. Figure 6 shows the O_2_ and H_2_ evolutions and the current density observed for each film on the time course of the experiment. As expected by the reaction stoichiometry, the molar amount of H_2_ produced doubles that of O_2_ within the experimental sensitivity of the gas-chromatographic analysis. Both SC films show similar performances, in correspondence with the Tafel plots and CV curves. A summary of the electrocatalytic tests and activity indicators of these three films is also reported in Table 1. The highest O_2_ evolution was obtained with ED*5c*, in agreement with the higher current densities. It is worth noting that the morphologies of the ED film and the SC film are different. In the first case, the MnO_x_ materials were grown forming a well-connected network of nanosheets that after calcination sintered to form interconnected nanorods, whereas the SC method yielded to non-highly associated particles. Therefore, it is expected that particles boundaries can generate an additional obstacle to charge transport in the SC films, as discussed above from the EIS data analysis, thus influencing their performance. The highest faradaic efficiencies (95% and 97% for O_2_ and H_2_ production, respectively) were measured with Mn_2_O_3_-SC, probably due to the higher crystallinity of the starting α-Mn_2_O_3_ powder material. In addition, in agreement with the previous results, the sample Mn_2_O_3_-SC-TT reported the lowest O_2_ and H_2_ faradaic efficiencies, confirming the incomplete transformation of the MnO_2_ phase.

## 4. Conclusions

This work compares the effectiveness of spin-coating (with polyethylene oxide as a binder) and electrodeposition as techniques for the preparation of stable MnO_x_-based electrodes active in the water oxidation reaction. Though spin-coating requires previous ball-milling of the catalyst in order to obtain stable and active films from originally microstructured powders, it leads to uniform films and allows removal of polyethylene oxide at mild temperatures (as low as 200 °C), and therefore it results a method potentially suitable to obtain electrodes of different types of catalysts.

Electrodeposition allows films to be obtained with nanostructured morphology, but the catalyst is amorphous, its catalytic activity depending on both deposition time and calcination. Concerning water oxidation, electrodeposited films indeed reached activities comparable (or higher) with respect to spin-coated films only after calcination at 500 °C, a treatment leading to the formation of α-Mn_2_O_3_, the active phase in spin-coated films as well. Calcination was particularly effective at intermediate electrodeposition times, but not at longer deposition times, suggesting that there is an upper limit to the thickness of the α-Mn_2_O_3_ film above which the overall exposed surface is not actually exploited.

Electrochemical impedance spectroscopy showed that electrodeposited films have better charge transport properties compared to spin-coated ones, as shown by a higher electrocatalytic activity of the former at high overpotential. On the other hand, at low overpotential, spin-coated films presented faster kinetics at the electrode/electrolyte interface, showing half of the charge transfer resistance compared to electrodeposited ones. Such result suggests that spin-coated films have better-defined electrocatalytic properties (at low applied potentials), likely due to the higher crystallinity of the pre-formed commercial catalysts. As a consequence, the spin-coating procedure presented here appears to be a simple and reliable method to fabricate electrodes with pre-synthesized powders of water oxidation catalysts.

## Figures and Tables

**Table 1:** Catalytic properties of the MnO_x_ films prepared by different methods.

Sample	On-set Potential ^a^ (V *vs.* RHE)	Tafel Slope (mV·dec^−1^)	Current Density at 2 V *vs.* RHE ^b^ (mA·cm^−2^)	O_2_ Production ^c^ (μmol)	Faradaic Efficiency for O_2_ Production (%)	Faradaic Efficiency for H_2_ Production (%)
Mn_2_O_3_-SC-TT	1.61	125	0.58	14	78	86
Mn_2_O_3_-SC	1.58	134	0.44	12	95	97
Mn_3_O_4_-SC	1.75	185	0.11	-	-	-
ED*5c*	1.58	115	1.25	48	85	85

^a^ Potential measured from the last CV cycle; ^b^ Current density measured from the last CV cycle; ^c^ Total O_2_ produced in 1 h under an applied potential of 2.0 V *vs.* RHE.

## Supplementary Materials

The following are available online at www.mdpi.com/1996-1944/9/4/296/s1. Figure S1: Cross-section FE-SEM image of a spin-coated film made with a non-ball-milled Mn_2_O_3_ powder; Figure S2: FE-SEM cross-section images of the films prepared by spin-coating of MnO_2_ (a); Mn_2_O_3_ (b) and Mn_3_O_4_ (c) powders; as-made electrodeposited 5-min film (d); Figure S3: UV-Vis transmittance spectra of the electrodeposited films: as-made (continuous line) and calcined at 500 °C (dotted line); Figure S4: Photographs of the as-made films prepared by electrodeposition at different deposition times; and Figure S5: Nyquist plots of the EIS measurements acquired using the α-Mn_2_O_3_-based electrodes at 1.6, 1.8 and 2.0 V *vs.* RHE.
