# Supplementary material for: Spin-Coated vs. Electrodeposited Mn Oxide Films as Water Oxidation Catalysts
Source: Materials (Basel). 2016 Apr 19;9(4):296. doi: 10.3390/ma9040296 (PMC5502989; doi:10.3390/ma9040296)
Supplement: Supplementary file 1 [file materials-09-00296-s001.pdf]

Supplementary Materials: Spin-Coated *vs.* Electrodeposited Mn Oxide Films as Water Oxidation Catalysts

Simelys Hernández, Carminna Ottone, Sara Varetti, Marco Fontana, Diego Pugliese, Guido Saracco, Barbara Bonelli and Marco Armandi

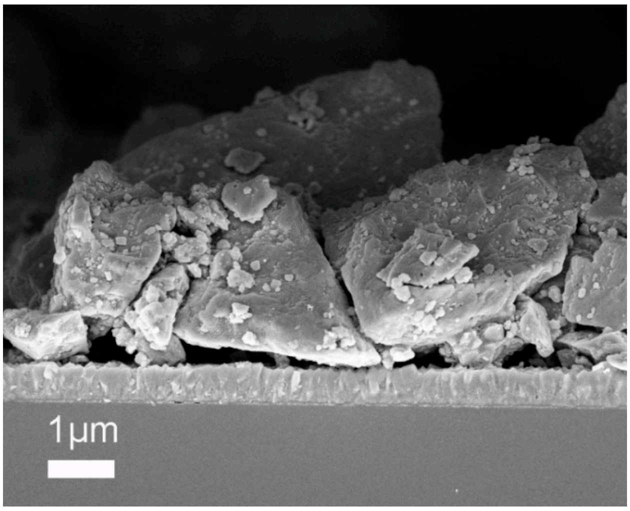

Figure S1. Cross-section FE-SEM image of a spin-coated film made with a non-ball-milled Mn_2O_3 powder.

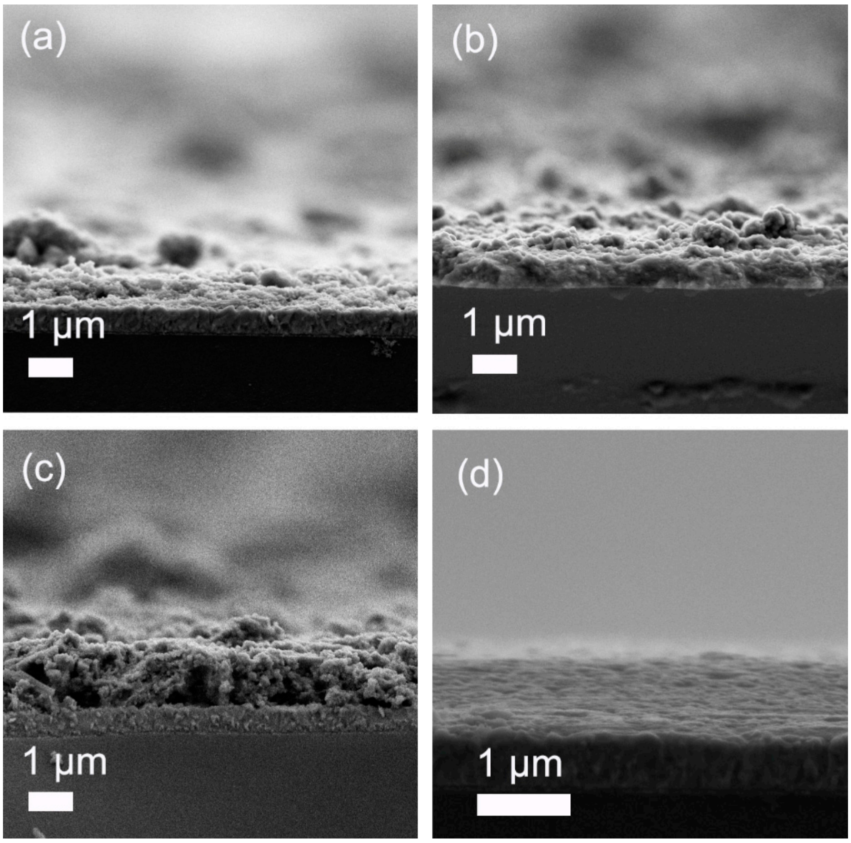

Figure S2. FE-SEM cross-section images of the films prepared by spin-coating of MnO_2 (**a**); Mn_2O_3 (**b**) and Mn_3O_4 (**c**) powders; as-made electrodeposited 5-min film (**d**).

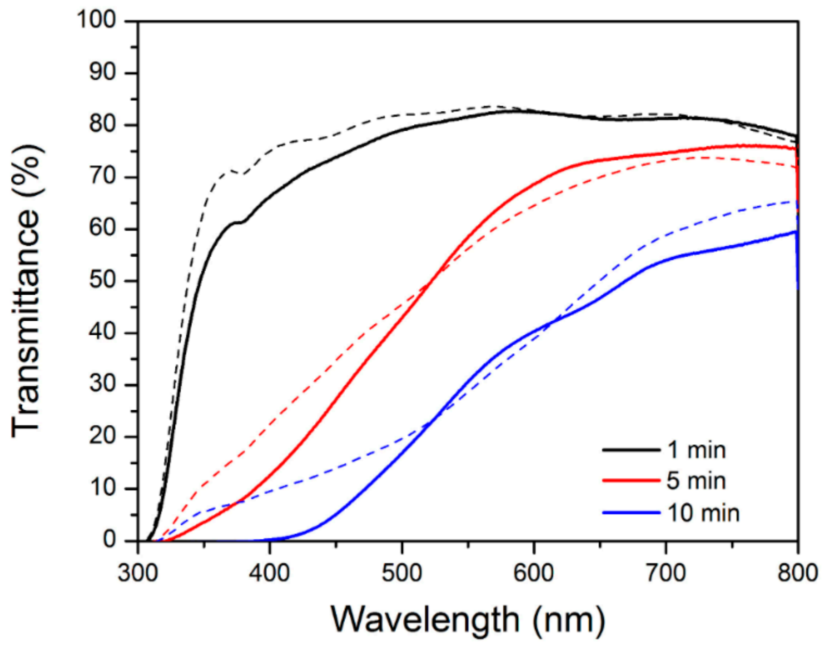

Figure S3. UV-Vis transmittance spectra of the electrodeposited films: as-made (continuous line) and calcined at 500 °C (dotted line).

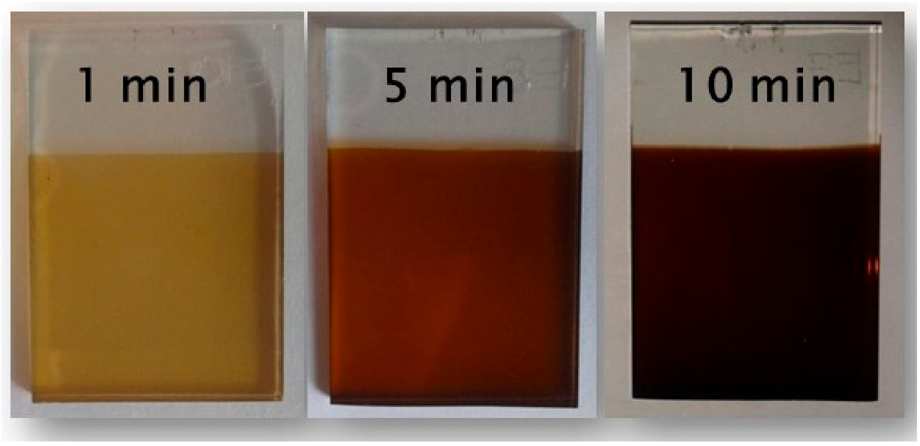

Figure S4. Photographs of the as-made films prepared by electrodeposition at different deposition times.

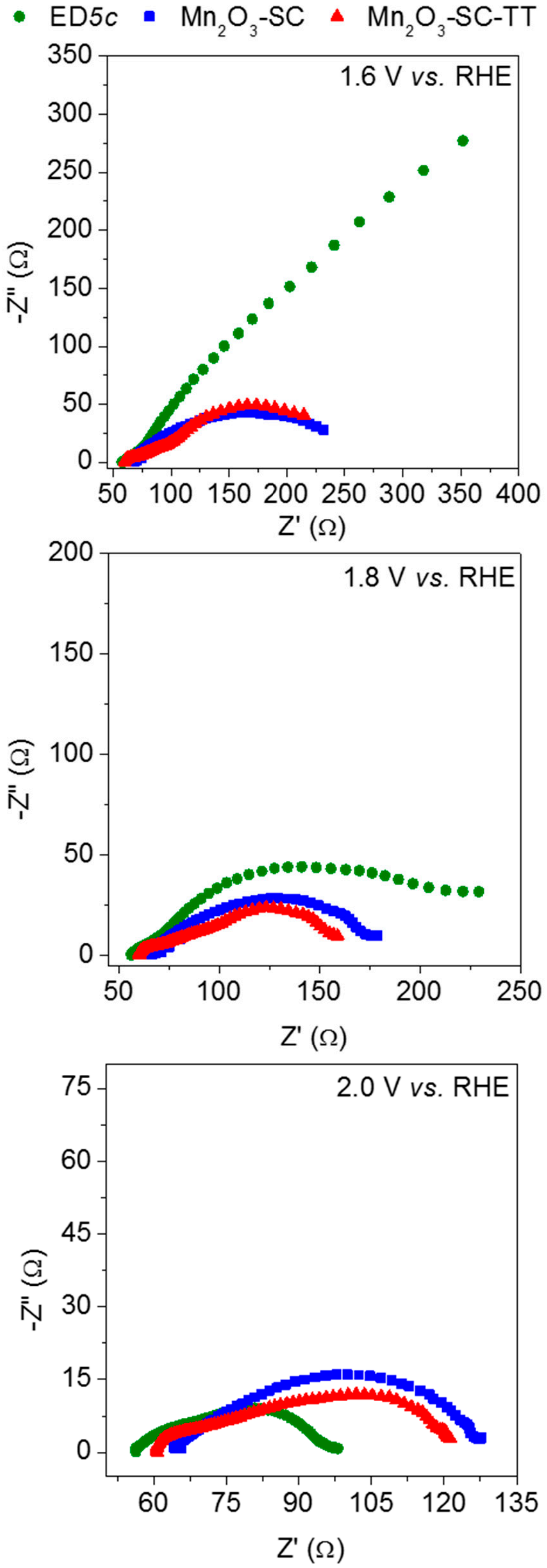

Figure S5. Nyquist plots of the EIS measurements acquired using the α-Mn_2O_3-based electrodes at 1.6, 1.8 and 2.0 V *vs*. RHE.
